# Reflections on Keywords: Definition, Life and Origin

**DOI:** 10.3390/life15091376

**Published:** 2025-08-29

**Authors:** André Brack

**Affiliations:** Centre de Biophysique Moléculaire, CNRS, 1 rue Charles Sadron, 45000 Orléans, France; andrebrack45@gmail.com

## 1. About Definition

‘Words, words, words”. This was Hamlet’s reply to Polonius’ question “What do you read, my lord?” By repeating the word three times, Hamlet suggests that what he is reading is meaningless. Meaningless for Hamlet, words are important for astrobiologists who try to decipher the origins of life on Earth and beyond. For them, “what are we looking for?” would be the question.

Would a definition of life be really helpful? Undoubtedly, scientists have to specify what they are trying to reproduce in the laboratory. The same applies when implementing a space mission to discover life beyond the Earth. As for mathematics, the primary role of a definition is to be useful in demonstration. But what demonstration can be expected when over 100 definitions of life exist, often in conflict with one another [1,2,3]? I am tempted to follow Jack Szostak in stating that “Attempts to define life do not help to understand the origin of life” [4], but attempts to understand the origin of life will likely eventually help in our ongoing endeavour to define life. It may be more judicious to describe life by listing the capabilities required rather than confining life into a rigid framework. In this respect, the chemist, through his laboratory experiments, approaches the question of origins without *a priori* convictions. Without unshakeable loyalty to a concept, hypotheses are postulated, which are then confirmed or refuted by experimental facts or demonstrations. In the absence of fossil traces of prebiotic events, he is searching, step by step, for the different types of functions that may have driven the chemistry of the first living systems.

## 2. About Life

Any open system capable of self-reproduction, i.e., making more of itself by itself, and also capable of evolving, will be considered as a living entity. As an open system, it necessarily receives energy and matter, which dispenses with including these requirements in the description. All replicating systems are, by definition, autocatalytic [5], but autocatalysis is not sufficient since the system must possess the capacity to cope with deleterious factors in its surroundings [6].

## 3. About Origin

The word is ambiguous since it can be understood as “origin = cause”, an in situ birth of life on Earth, or as “origin = beginning”, a point of inception or an emergence, which could as well include panspermia, the interplanetary transfer of life, thus pushing the origin of life behind the scene without deciphering it. Although it is still difficult to prove that life was transported to the Earth, experimental support has been provided for the different steps of the process, the escape from the parent body [7], the travel conditions in space [8,9], and the non-destructive deposition of the biological material on another planet [10].

Was the origin of life rapid or excessively slow? Numerous estimates have been proposed, generally spanning several hundred million years, a time interval supposed to separate two impacts capable of sterilizing the primitive Earth, the number of which may have been between 0 and 6 [11]. In fact, it depends on what is meant by “origin of life”. Should we simply consider the start of the first autocatalytic and evolving organized molecular systems from the elements, or should we include the time necessary for the manufacture of the elements? The start-up can be very brief, or even instantaneous, like supersaturated solutions that crystallize almost instantly upon the addition of crystals serving as seeds. Providing the basic elements can take much longer. How much? Nobody knows, although analysis of micrometeorites collected in Antarctica shows that space provided enormous quantities of raw carbonaceous material to the primitive Earth during the early phase of intense bombardment [12].

Was the origin of life banal or highly improbable? The original chemical structure had to be robust enough to survive meteorite impacts and possibly restart after larger impacts. It is reasonable to think that, in order to be robust, primitive life had to be relatively simple and capable of being repeated several times. The discovery of other examples of life on other celestial bodies would confirm the relative simplicity of the origin of life by providing proof of its repetitive nature.
